# Multivariable Analysis of Correlation Between Anatomical Features of Mandibular Third Molars and Pericoronitis

**DOI:** 10.1155/ijod/8260559

**Published:** 2024-12-12

**Authors:** Bao-Ngoc Thi Nguyen, Chi-Tam Nguyen-Le, Bich-Ly Thi Nguyen, Son Hoang Le

**Affiliations:** Department of Oral Surgery, Faculty of Odonto-Stomatology, University of Medicine and Pharmacy at Ho Chi Minh City, Ho Chi Minh City, Vietnam

**Keywords:** mandibular third molar, Pell–Gregory classification, pericoronitis, risk factors, winter classification

## Abstract

**Objective:** The correlation between anatomy features of impacted mandibular third molars (M3Ms) and prevalence of pericoronitis was only interpreted using univariate analysis. This study investigated this correlation using multivariable analysis to determine the relationship between pericoronitis prevalence and the M3Ms' anatomical features.

**Methods:** This cross-sectional study recruited 245 patients with 338 impacted M3Ms. One researcher collected participants' demographic characteristics such as sexes, age, side, and pericoronitis condition. The radiographic characteristics of M3Ms, including eruption direction and impacted levels according to Pell–Gregory classification, were assessed based on their orthopantomograms. Initially, univariate analyses were used to determine potential demographic and radiographic factors that correlated to pericoronitis. These factors were, then, analyzes using Firth's logistic regression.

**Results:** No significant difference was found between non- and pericoronitis groups about sexes, age, side (*p*  > 0.05). The univariate analyses showed that proportion of vertical impacted levels and eruption direction of M3Ms were significantly different between non- and pericoronitis groups. Firth's logistic regression analysis indicated that M3Ms with impacted level A were more likely to suffer from pericoronitis than ones at level B (odds ratio (OR) = 3.34), wheraes M3Ms impacted level II had higher risk of pericorinitis than ones at level I (OR = 1.63). Vertical M3Ms were more likely to develop pericoronitis than horizontal (OR = 5.78) ones.

**Conclusion:** M3M angulation and impacted level are significant factors relating to pericoronitis prevalence. M3Ms with vertical eruption, vertically level A, and horizontally level B are more likely to have pericoronitis.

## 1. Introduction

Pericoronitis is one of the most common diseases that are caused by impacted mandibular third molars (M3Ms), with the prevalence up to 82.41% [1]. Generally, its clinical symptoms are only limited as local inflammation such as pain, redness, and swelling in gingival tissue. However, in some individuals, the infection condition may gradually extend to surrounding tissues and exist simutaniously with other systemic symptoms, such as fever, lymphadenopathy, and upper respiratory tract infections [2]. Because these symptoms can significantly impact patients' quality of life, removing M3Ms that are likely to develop pericoronitis helps to prevent the existence of this disease [3, 4].

M3M accounted for the majority of pericoronitis cases reported in the medical literature. This is the result of the impacted condition which increase the uptake of food debris in the operculum [5]. It creates favorable conditions for bacterial growth and leads to host inflammatory reaction. Notably, the pericoronitis prevalence are not similar between different impacted condition [6]. Therefore, certain M3M anatomical features may provoke pericoronitis.

Some previous studies investigated the relationship of M3M anatomical features and pericoronitis prevalence [7–11]. However, their results were different which may be due to the statistical analysis method. Although these features may be intercorrelated with each other and lead to confounding results, these relationships were only analyzed using univariate analysis [12, 13]. This study aimed to interpret the relationship between M3M anatomical features and pericoronitis using a multivariable analysis.

## 2. Materials and Methods

### 2.1. Study Setting and Participants

This was a cross-sectional study of patients who visited the Department of Oral Surgery, Faculty of Odonto-Stomatology, University of Medicine and Pharmacy at Ho Chi Minh City for M3M extractions. The study participants were recruited between December 2022 and March 2024. The Biomedical Research Ethics Council of the University of Medicine and Pharmacy at Ho Chi Minh City (No. 22491) approved the study protocol and informed consent was obtained from all participants.

The inclusion criteria for participants were as follows: (1) full permanent dentition, except for third molars, and (2) orthopantomograms (OPGs) clear enough to assess M3Ms and their surrounding structures. The exclusion criteria were as follows: (1) pregnancy; (2) mental stress, upper respiratory tract infection, and menstruation; (3) smoking; and (4) systemic diseases that may relate to pericoronitis.

### 2.2. Sample Size Calculation

The recruited M3M was determined based on the equation for estimating prevalence with absolute error: $n\geq Z_{1-\frac{\alpha }{2}}^{2}\frac{p\left(1-p\right)}{d^{2}}.$

According to Ye et al. [1], the prevalence of M3M pericoronitis was 82.41%. The sample size was calculated as *n* ≥ 223.

### 2.3. Data Collection

Initially, a researcher (Bao-Ngoc Thi Nguyen) collected the informed consent and demographic characteristics (age, sex, side, and pericoronitis condition) of the participants. M3M that was diagnosed as pericoronitis must have at least one of the following symptoms in the surrounding soft tissue: pain, redness, swelling, and purulent discharge. The radiographic characteristics of the M3Ms were analyzed and measured twice by a single researcher (Chi-Tam Nguyen-Le) using NanoCAD (version 5.0, Nanosoft AS, Lillestrøm, Norway). The collected variables on OPGs were as follows: (1) Winter classification based on the angle between M3Ms and the adjacent mandibular second molars (M2Ms); (2) M3M impacted levels in the horizontal and vertical directions according to the Pell–Gregory classification; (3) M3M mesiodistal dimension; and (4) M3M eruption space. For the interval variables, the final data are the mean values of the two measurements. For categorical variables, the data were identical for both the assessments.

### 2.4. Statistical Analysis

Data were analyzed using SPSS (version 24.0; IBM Corp., Armonk, NY, USA). Demographic and radiographic characteristics of the sample were described as means ± standard deviations (SDs) for interval variables, and ordinal and nominal variables were described with counts and percentages. Independent samples *t*-tests, *χ*^2^ tests, and Fisher's exact test were used to compare differences between the pericoronitis and nonpericoronitis groups. Based on these statistical results, variables that showed a certain correlation with pericoronitis (*p* < 0.25) were included in the Firth's logistic regression analysis. Significant factors were reported as odds ratios (ORs) and 95% confidence intervals (CIs). Statistical significance was set at *p*  < 0.05.

## 3. Results

This study recruited 338 M3Ms from a total of 245 patients. Among them, 146 (43.2%) were diagnosed pericorontitis.  Table 1 shows the demographic and radiographic characteristics of the M3Ms.

### 3.1. Characteristics of M3Ms With and Without Pericoronitis

Table 2 shows the demographic characteristics of pericoronitis M3Ms and their counterparts. Generally, no significant differences in the demographic characteristics were found between the pericoronitis and nonpericoronitis groups.

**Figure 1:**
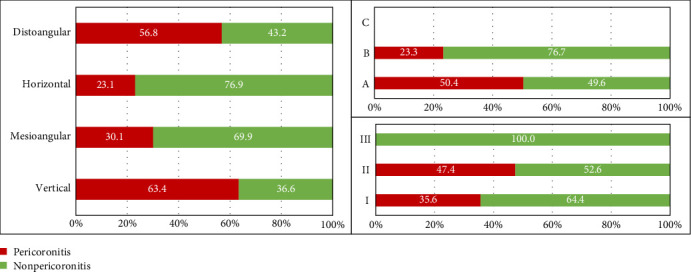
Proportion of M3Ms with and without pericoronitis according to angulation types and Pell–Gregory classification. M3M, mandibular third molar.

Figure 1 shows the percentage of pericoronitis M3Ms according to the Winter and Pell–Gregory classification. The percentage of pericoronitis teeth was significantly different between the erupted directions (*χ*^2^ = 35.96, *p*  < 0.001). This percentage was highest in the vertical M3Ms and lowest in the horizontal ones. At the vertical impact level, the percentage of pericoronitis was significantly different between levels A and B (*χ*^2^ = 19.72, *p*  < 0.001). Fisher's exact test indicated significant association between the horizontal impact levels and pericoronitis condition (*p*=0.024).

### 3.2. Firth's Logistic Regression Analysis of Risk Factors for Pericoronitis

Table 3 shows that vertical M3Ms were more likely to develop pericoronitis than horizontal teeth (OR = 5.78; 95% CI = 2.12–15.63). For mesio- and distoangular M3Ms, no significant effect on the likelihood of pericoronitis was observed. The M3Ms at vertically impacted level A were more likely to suffer from pericoronitis than those at level B (OR = 3.34; 95% CI = 1.93–5.78). In addition, the M3Ms at horizontally impacted level II were more likely to have pericoronitis than teeth at level I (OR = 1.63; 95% CI = 1.01–2.63).

## 4. Discussion

Pericoronitis is an inflammatory condition of the soft tissue surrounding impacted M3Ms. Previous studies indicated that eruption status and impacted level of M3Ms significantly related to risk of pericoronitis [6]. However, the results vary and may be confounded by risk factors. Firth's logistic regression analysis was used to control for the latent correlation between potential risk factors. The results revealed that certain erupted directions and vertically impacted levels of M3Ms can increase the risk of pericoronitis.

**Figure 2:**
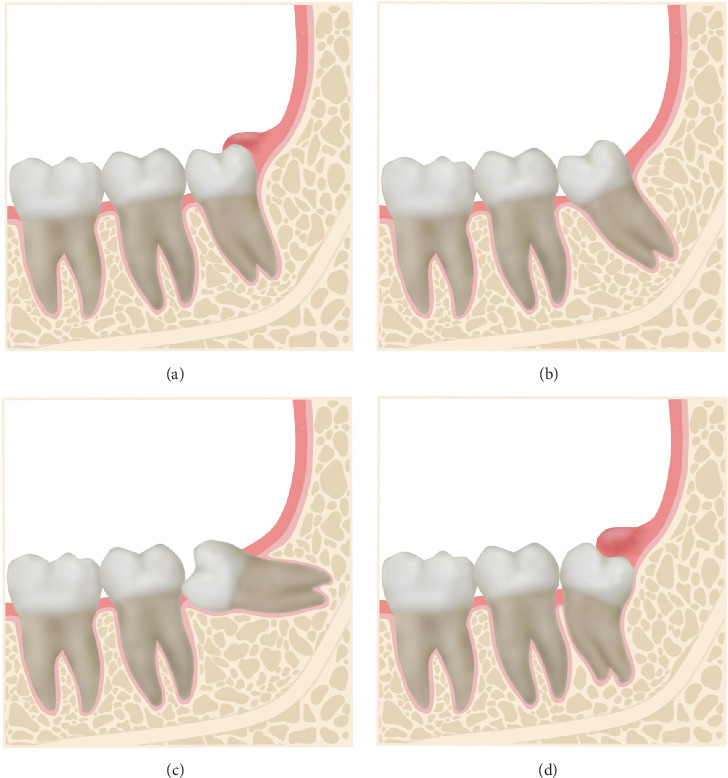
Illustration of different M3M angulation types with the same Pell–Gregory classification (IA) and distal operculum. (A) Vertical, (B) mesioangular, (C) horizontal, and (D) distoangular. Although the four M3M types have the same Pell–Gregory classification, vertical and distoangular teeth are more likely to have an operculum covering the crown. M3M, mandibular third molar.

This study showed that the prevalence of pericoronitis was significantly different among eruption directions. Specifically, this prevalence was the highest in vertical M3Ms and increased the risk 5.78 times more than the horizontal ones. Although no significant risk was found for distoangular M3Ms, its prevalence of pericoronitis was notably higher than those of mesioangular and horizontal teeth. This finding is aligned with most of previous studies that reported notably higher prevalence of pericoronitis in vertical and distoangular M3Ms [7, 11]. Other studies that recruited only pericoronitis M3Ms also showed the dominance of vertical and distoangular teeth in their samples [9, 14, 15]. To the best of our knowledge, there is no persuasive explanation for the effect of eruption direction on pericoronitis. We hypothesized that in vertical and distoangular M3Ms, the occlusal surface contacts the overlying soft tissue. Therefore, food debris can become trapped between these tissues, causing pericoronitis (Figure 2). Conversly, in mesial and horizontal ones, the accumulation of food is more likely to cause dental caries at the distal surface of mandibular second molars [16]. A few studies stated that mesioangular M3Ms had more chance to develop this inflammatory condition than other M3Ms [10, 17]. However, recruiting only pericoronitis M3Ms may underestimate the actual effect of eruption directions because different angulations do not share the same proportion among the impacted M3Ms.

Firth's logistic regression analysis showed that the vertical impact level of M3Ms affected the prevalence of pericoronitis. Specifically, impacted M3Ms at level A have 3.34 times higher chance to have pericoronitis than teeth at level B. Similarity, previous studies revealed that the majority of pericoronitis M3Ms were classified level A according to Pell–Gregory classification [8, 18]. Some hypotheses explain the effect of vertical impacted levels. First, the M3Ms level A reached the same position or higher than the occlusal plane. Consequently, they are more likely to have trauma due to the opposite tooth eruption [11]. Second, this high position also makes them prone to food debris and plaque accumulation and triggers pericoronitis. Additionally, M3Ms at level B were partially covered by soft tissue and bone more than those at level A. This condition could also reduce the presence of soft tissue pockets and prevalence of pericoronitis. A previous study reported that M3Ms at level B were far more common that the other two levels in recruited pericoronitis cases [17]. However, this result could not prove that M3Ms at level B were more likely to cause this disease.

The results showed that the prevalence of pericoronitis in M3Ms with horizontal levels I and II was approximately one-fourth that of the recruited patients. However, this number in level III M3Ms was zero, which may be due to the total coverage of the wisdom teeth by the ramus. Although statistical analysis did not show any significant differences between horizontal impacted levels, we noted that M3Ms' exposure to the oral cavity clearly changed disease prevalence. Previous studies used either Pell–Gregory or other classifications also supported this finding [8, 14, 15, 18].

The use of multivariable logistic regression to interpret the statistical data is the most outstanding point of this study. This helped reduce the confounding issue between the potential factors that may affect the pericoronitis condition, such as erupted direction and impacted level in the vertical and horizontal dimensions. However, this study design has some limitations that can be addressed in future studies. First, the M3Ms position was determined only on OPGs that lacked the three-dimensional assessent. Advancing medical imaging techniques such as cone-beam computed tomography and magnetic resonance imaging can overcome this issue. Second, clinical judgment can notice impacted level of M3M under soft tissue more accurate than OPG assessment. Third, the number of M3Ms of type III and C was very limited (less than 2%). Although their proportions in this study were similar with those of the previous ones, larger samples can increase the number of cases and improve the final models [19].

## 5. Conclusions

Within the limitations of this study, it was revealed that M3Ms angulation and anatomical position significantly affected the prevalence of pericoronitis. Vertical M3Ms were more likely to have pericoronitis than horizontal ones. In vertical impacted levels, M3Ms at level A and were more associated with pericoronitis than those at level B. In addition, M3Ms at level II had higher risk than those at level I to suffer from pericoronitis.

## Figures and Tables

**Table 1:** Demographic and radiographic characteristics of the study sample.

Characteristics	*n*	%
Sex *n* (%)		
Female	200	59.2
Male	138	40.8
Side *n* (%)		
Left	167	49.4
Right	171	50.6
Winter classificatio*n*/*n* (%)		
Vertical	101	30.0
Mesioangular	166	49.3
Horizontal	26	7.7
Distoangular	44	13.1
Horizontal impacted level, *n* (%)		
I	104	30.8
II	230	68.0
III	4	1.2
Vertical impacted level, *n* (%)		
A	248	73.4
B	90	26.6
C	0	0.0

*Note: N* = 338. Ages were on average 22.6 years old (SD = 3.7).

Abbreviation: SD, standard deviation.

**Table 2:** Comparision of demographic characteristics between non- and pericoronitis groups.

Characteristics	Pericoronitis *N* = 146	Nonpericoronitis *N* = 192	*χ* ^2^/t	*p*-Value
Sex^a^, *n* (%)	—	—	2.890	0.089
Female	94 (47.0)	106 (53.0)	—	—
Male	52 (37.7)	86 (62.3)	—	—
Age^b^, mean (SD), year	22.9 ± 3.9	22.4 ± 3.6	1.254	0.211
Side^a^, *n* (%)	—	—	1.272	0.259
Left	67 (40.1)	100 (59.9)	—	—
Right	79 (46.2)	92 (53.8)	—	—

Abbreviation: SD, standard deviation.

^a^ = *χ*^2^ test.

^b^ = Independent samples *t*-test.

**Table 3:** Firth's logistic regression analysis of risk factors of pericoronitis.

Variable	Estimate	SE	Lower CI	Upper CI	*p*-Value
Constant	−1.86	0.88	−3.58	−0.18	0.030
Sex^a^					
Female	0.04	0.26	−0.46	0.53	0.887
Age	0.03	0.03	−0.03	0.09	0.354
Winter classification^b^					
Mesioangular	0.22	0.51	−0.73	1.28	0.658
Vertical	1.53	0.53	0.54	2.62	0.002
Distoangular	0.94	0.57	−0.13	2.11	0.087
Vertical impacted level^c^					
B	−1.06	0.30	−1.66	−0.50	<0.001
Horizontal impacted level^d^					
II	0.66	0.28	014	1.21	0.013
III	−0.96	1.70	−5.87	1.40	0.485

*Note: N* = 338.

Abbreviations: CI, confidence interval; SE, standard error.

^a^Reference = male.

^b^Reference = horizontal.

^c^Reference = A.

^d^Reference = I.

## Data Availability

The data that support the findings of this study are available on request from the corresponding author. The data are not publicly available due to privacy or ethical restrictions.
